# Normative data of ultrasound quadriceps rectus femoris geometric values. The ECOSARC-2 Study

**DOI:** 10.1016/j.jnha.2025.100608

**Published:** 2025-06-17

**Authors:** Ester López Jiménez, Paola Reinoso Párraga, Alfonso González Ramírez, Marta Neira Álvarez, Adriana Abizanda Saro, Marisa Fernández González de la Riva, Cristina Alonso Bouzón, Victoriano Chavero Carrasco, Gema Montemayor Galán, Juan Rodríguez Solís, Rubén Alcantud Córcoles, Raquel Ramírez Martín, Carmen de Pablos Hernández, Fabricio Zambom-Ferraresi, Brian Dax Vásquez, Pedro Luis Sagüillo González, Ignacio Morón Merchante, Rafael García Molina, Rocío Menéndez Colino, Nicolás Martínez Velilla, Fernando Andrés Pretel, Elena Gómez Jiménez, Marta Sáez Blesa, Elisa Belén Cortés Zamora, Almudena Avendaño Céspedes, Pedro Abizanda

**Affiliations:** a Geriatrics Department, Complejo Hospitalario Universitario de Albacete, Albacete, Spain; b Geriatrics Department, Hospital Universitario La Paz, Research Institute la Paz (IdiPAZ), Madrid, Spain; c Geriatrics Department, Hospital Universitario de Salamanca, Spain; d Geriatrics Department, Hospital Universitario Infanta Leonor, Fundación para la Investigación e Innovación Biomédica H.U. Infanta Leonor y H.U. Sureste, Madrid, Spain; e Centre d´Atenciò Primària Sant Martí de Provençals, Barcelona, Spain; f Navarrabiomed, Hospital Universitario de Navarra (HUN), Universidad Pública de Navarra (UPNA), Spain; g Geriatrics Department, Hospital Universitario de Getafe, Madrid, Spain; h CIBER de Fragilidad y Envejecimiento Saludable (CIBERFES), Instituto de Salud Carlos III, Madrid, Spain; i Centro de Salud de Valdefuentes, Cáceres, Spain; j Centro de Salud Universitario Goya, Madrid, Spain; k Geriatrics Department, Hospital Universitario de Guadalajara, Spain; l Centro de Salud Arroyo de la Luz, Cáceres, Spain; m Universidad Autónoma de Madrid, Spain; n Statistics Department, Complejo Hospitalario Universitario de Albacete, Spain; o Escuela de Enfermería de la Fundación Jiménez Díaz, Universidad Autónoma de Madrid, Spain; p Facultad de Medicina de Albacete, Universidad de Castilla-La Mancha, Albacete, Spain

**Keywords:** Ultrasonography, Muscle, Normative values, Sarcopenia, Older adults

## Abstract

**Objectives:**

To describe geometric muscle ultrasound normative values of Quadriceps Rectus Femoris (QRF) at mid-thigh in Spanish community-dwelling older adults. Secondary objectives included evaluating the associations between ultrasound measurements and physical function, muscle strength, frailty, and dependency in basic activities of daily living.

**Design:**

Cross-sectional study.

**Setting:**

Outpatient clinics of Geriatric Departments at seven University Hospitals and three Primary care settings of Spain.

**Participants:**

424 community-dwelling persons ≥70 years old.

**Measurements:**

Ultrasound measurements were acquired using a Clarius® L7 HD3 probe. QRF thickness (QRFT), QRF area (QRFA), subcutaneous adipose thickness (SAT), total mid-thigh thickness (TTT), and pennation angle (PA) were calculated. Normative values are provided, and associations with comorbidity, dependency, strength, frailty and physical function are described.

**Results:**

Mean age was 81.5 years (range 70−99), 66.7% were female. Mean Barthel index was 93 (SD 11), and mean FRAIL score was 1.1 (1.2). Mean SPPB was 8.7 (SD 2.9) and mean grip strength was 20.7 (8.0). Median values of QRFT, QRFA, SAT, TTT, and PA for women and men were 14.2 / 16.1 mm, 5.4 / 6.4 cm^2^, 15.1 / 9.2 mm, 41.1 / 40.1, and 10.0 / 10.6 respectively. Functional variables were strongly associated with QRFT, QRFA, and TTT in men, and PA with SPPB in both sexes.

**Conclusions:**

The ECOSARC-2 study provides normative values of geometric mid-thigh muscle ultrasound parameters in community-dwelling older adults. These values are essential for determining sex-specific cut-off points for conditions such as sarcopenia, frailty, malnutrition, and for predicting relevant outcomes.

## 1 Introduction

Sarcopenia is a significant health concern among older adults, associated with numerous adverse outcomes, including increased mortality, higher risks of falls and fractures, functional decline, reduced mobility, greater rates of hospitalization and institutionalization, and a substantial deterioration in quality of life [[1], [2], [3], [4]]. Additionally, sarcopenia imposes a considerable economic burden due to increased healthcare costs [5]. Epidemiological studies estimate the overall prevalence of sarcopenia to be approximately 10% in both men and women [6], indicating that a significant proportion of older adults, including those who are otherwise healthy, may be affected [7]. These findings highlight the urgency of addressing sarcopenia as a critical public health issue and underscore the need for further research into effective prevention strategies and treatment approaches to mitigate its impact on aging populations.

Muscle ultrasound has emerged as a promising tool for evaluating the musculoskeletal system. It is safe, cost-effective, reliable, valid, and rapid, making it particularly suitable for assessing muscle quality and quantity in the context of sarcopenia diagnosis and management [[8], [9], [10], [11]]. However, despite these advantages, muscle ultrasound faces challenges such as high methodological heterogeneity and a lack of standardization. The absence of consensus on diagnostic thresholds has limited its widespread clinical adoption for sarcopenia assessment [12]. Nevertheless, ultrasound-derived parameters have demonstrated strong correlations with muscle strength and functional capacity and provide valuable prognostic insights into sarcopenia [13].

Further research is essential to identify key muscles and the most accurate ultrasound measurements for detecting individuals with sarcopenia or those at risk of developing it [14]. Standardized protocols and well-defined diagnostic cut-off values are urgently needed to improve the clinical utility of ultrasound in this context [15]. Among the muscles studied for sarcopenia assessment, the quadriceps rectus femoris (QRF) stands out as particularly promising. However, studies specifically examining QRF ultrasound measurements for diagnosing sarcopenia remain limited [[16], [17], [18]], especially in community-dwelling older adults. Furthermore, the lack of normative data for this population limits comparisons across global cohorts. Establishing such normative data is crucial for understanding muscle architecture in older adults and defining diagnostic cut-off values.

To address these gaps in knowledge and advance the field of sarcopenia assessment using ultrasound technology, the ECOSARC-2 study was designed and conducted. This study aims to provide critical insights into QRF measurements in community-dwelling older adults while contributing to the development of standardized diagnostic criteria.

## 2 Methods

The ECOSARC-2 study is a cross-sectional study conducted between January and December 2024. Participants were recruited from outpatient clinics across seven University Hospitals: Complejo Hospitalario Universitario de Albacete (n = 74), Hospital Universitario La Paz in Madrid (n = 61), Hospital Universitario de Salamanca (n = 55), Hospital Universitario Infanta Leonor in Madrid (n = 46), Hospital Universitario de Navarra (n = 40), Hospital Universitario de Getafe (n = 38), and Hospital Universitario de Guadalajara (n = 16). Additionally, participants were enrolled from three Primary Care settings: Metropolitana Nord in Barcelona (n = 41), Gerencia de Atención Primaria de Cáceres (n = 35), and Centro de Salud Goya in Madrid (n = 18). The project received initial approval from the Ethics Committee of Albacete (Number 2023-082; 27/June/2023), followed by approval from the Ethics Committees of the other nine participating sites. Informed consent was obtained from all participants.

The main objective of the ECOSARC-2 study was to establish normative values for geometric ultrasound measurements of the quadriceps rectus femoris (QRF) in community-dwelling older adults. Secondary objectives included evaluating the associations between ultrasound measurements and physical function, muscle strength, frailty, and dependency in basic activities of daily living (BADL).

The inclusion criteria for the ECOSARC-2 study were women and men aged 70 years or older with a Holden's Functional Ambulation Classification Scale score of level 1 or higher, excluding nonfunctional ambulator older adults (level 0). Exclusion criteria included an estimated life expectancy of less than 6 months, congenital or non-congenital absence of lower limbs, severe dementia, any factor impairing the ability to understand and sign informed consent in the absence of a caregiver, and any condition that, in the researchers' opinion, would prevent completion of the required study assessments. The sample size was initially calculated for 400 participants; however, a total of 424 individuals were ultimately included.

Sociodemographic data, body mass index (BMI), comorbidity using the Charlson index, frailty status assessed using both the frailty phenotype and the FRAIL instrument, hand grip strength measured with a JAMAR dynamometer, physical function evaluated through the Short Physical Performance Battery (SPPB), and dependency in basic activities of daily living assessed using the Barthel Index were collected.

### 2.1 Training of the sonographers

The training of sonographers for the ECOSARC-2 study involved a structured process to ensure consistency and accuracy in ultrasound measurements. Two geriatricians from each participating site received initial training from expert sonographers at the reference site. This training included a five-hour on-site session using the Clarius® L7 HD3 probe, accompanied by comprehensive training materials. Following this, the trainees practiced independently at their respective sites for one month, during which they could consult with the experts as needed.

Subsequently, an in-person agreement session was conducted with volunteer older adults to assess concordance between the researchers and the expert sonographers. Eight sonographers achieved a Pearson correlation coefficient of ≥0.900 with the expert and were directly certified. The remaining ten sonographers underwent an additional two-hour online training session before receiving final certification.

### 2.2 Ultrasound geometric muscle acquisition

**Fig. 1:**
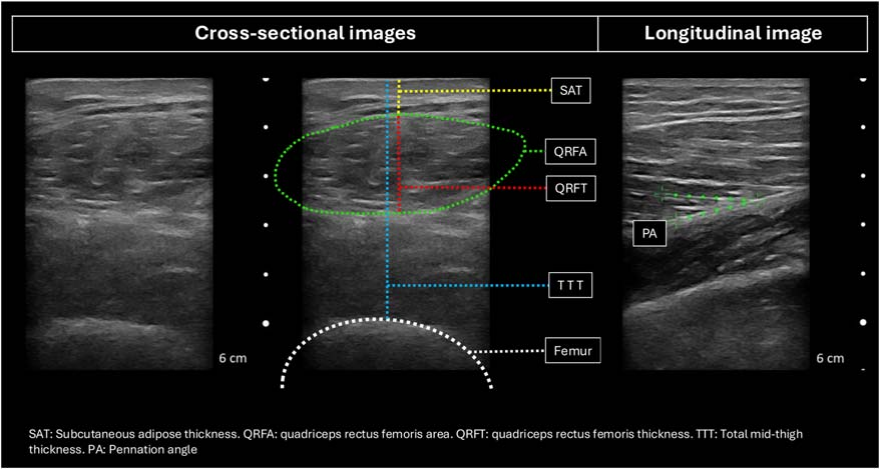
Ultrasound geometric muscle measurements.

Ultrasound measurements were performed at the mid-thigh of participants while lying in a supine position, both at rest and in a functional position, using a Clarius® L7 HD3 probe (Clarius Mobile Health, Vancouver, Canada). Three cross-sectional images of each leg were obtained, and the following geometric parameters were measured: quadriceps rectus femoris thickness (QRFT) in millimeters (mm), quadriceps rectus femoris area (QRFA) in square centimeters (cm²), subcutaneous adipose tissue thickness (SAT) in mm, and total mid-thigh thickness (TTT) in mm (Fig. 1). Additionally, three longitudinal images were acquired at the same site to measure the pennation angle (PA). PA is the angle between the muscle fascicle and the echo of deep aponeurosis in longitudinal images and has been involved in the understanding of skeletal muscle biomechanics and muscle force generation [19,20]. The mean values of the three measurements for each leg, as well as the mean values of both legs combined, were calculated and used as primary variables [21].

### 2.3 Statistics

Mean values, along with standard deviation (SD), range, and percentiles (P20, P25, P33, P50, P66, P75, and P80), were calculated for each ultrasound geometric variable separately for women and men. We chose an empirical non-parametric distribution without making any assumptions about the distribution, resulting in percentiles as the main data presentation form. The associations between muscle ultrasound measurements and functional variables were analyzed using correlation analyses and linear regression models.

## 3 Results

Table 1 summarizes the characteristics of the complete sample population and the sex groups. The mean age of the total sample was 81.5 years (range: 70–99), with 283 participants (66.7%) being female. The mean body mass index (BMI) was 27.3 kg/m^2^. Participants exhibited low comorbidity (mean Charlson Index: 1.3) but reported a high number of regularly consumed medications (mean: 7.5). Dependency in basic activities of daily living (BADL) was minimal (mean Barthel Index: 93), although physical function was moderately impaired (mean Short Physical Performance Battery [SPPB]: 8.7). Frailty status fell within the pre-frail range based on both the frailty phenotype (mean number of frailty criteria: 1.8) and the FRAIL instrument (mean FRAIL criteria: 1.1). The mean hand grip strength was 20.7 kg. Statistically significant differences were observed between women and men in comorbidity, dependency in BADL, physical function, frailty, and grip strength.

**Table 1:** Descriptive characteristics of the sample.

	Complete sample			Men (n = 141)		Women (n = 283)	
	n	Mean (SD)	Range	Mean (SD)	Range	Mean (SD)	Range
Age	424	81.5 (6.2)	70−99	81.6 (6.3)	70−97	81.4 (6.1)	70−99
BMI	414	27.3 (4,6)	16.4−46	27.2 (3.8)	18.8−40.0	27.4 (5.0)	16.4−46.0
Charlson index	417	1.3 (1.6)	0−10	2.0 (2.0)‡	0−10	1.0 (1.3)‡	0−6
Number of drugs	411	7.5 (3,9)	0−22	7.4 (3.9)	0−19	7.6 (4.0)	0−22
Barthel index	424	93 (11)	45−100	94 (10)†	50−100	92 (11)†	45−100
SPPB	422	8.7 (2.9)	0−12	9.3 (2.7)‡	3−12	8.4 (3.0)‡	0−12
Fried criteria	422	1.8 (1.4)	0−5	1.5 (1.3)†	0−5	1.9 (1.5)†	0−5
Grip strength	402	20.7 (8.0)	5−51.4	27.8 (8.2)‡	9.0−51.4	17.1 (5.0)‡	5.0−30.5
FRAIL scale	423	1.1 (1.2)	0−5	1.0 (1.1)*	0−4	1.2 (1.2)*	0−5

BMI: Body mass index. SPPB: Shosr Physical Performance Battery. * p < 0.05. † *p* < 0.01. ‡ *p* < 0.001.

Table 2 provides normative values for the five geometric muscle ultrasound variables. As anticipated, women had higher values for subcutaneous adipose tissue thickness (SAT) and total mid-thigh thickness (TTT), while men exhibited greater quadriceps rectus femoris thickness (QRFT), area (QRFA), and pennation angle (PA). However, differences in QRFT, TTT, and PA between sexes were relatively minor.

**Table 2:** Normative values of geometric ultrasound values of mid-thigh.

	n	Min	Max	Mean	SD	P 20 / 25 / 33 / 50 / 66 / 75 / 80
SAT men	138	3.0	28.7	9.8	3.9	7.0 / 7.3 / 7.9 / 9.2 / 10.2 / 11.3 / 12.2
SAT women	281	2.5	42.2	15.9	7.2	9.5 / 10.5 / 12.3 / 15.1 / 18.0 / 20.0 / 21.3
QRFT men	139	6.9	26.3	16.5	4.0	13.4 / 13.8 / 14.4 / 16.1 / 18.1 / 19.5 /20.2
QRFT women	280	7.8	25.3	14.4	2.9	11.9 / 12.3 / 13.1 / 14.2 / 15.6 / 16.2 / 17.0
TTT men	139	16.6	72.2	40.2	10.0	32.5 / 33.5 / 35.1 / 40.1/ 43.5 / 47.3 / 49.5
TTT women	282	16.6	78.3	42.5	10.7	33.5 / 35.9 / 37.9 / 41.1 / 45.5 / 48.8 / 51.2
QRFA men	139	2.6	13.3	6.5	1.9	4.9 / 5.2 / 5.6 / 6.4 / 7.1 / 7.4 / 7.9
QRFA women	282	1.8	10.9	5.3	1.4	4.2 / 4.3 / 4.7 / 5.4 / 5.9 / 6.2 / 6.4
PA men	132	4.4	21.8	10.9	3.0	8.5 / 8.9 / 9.4 / 10.6 / 11.9 / 12.9 / 13.3
PA women	282	2.9	19.6	10.1	2.9	7.6 / 8.2 / 8.9 / 10.0 / 11.2 / 11.8 / 12.3

n: number of participants with values. Min: lower value. Max: higher value. SD: standard deviation. P: percentiles. SAT: Subcutaneous adipose tissue. QRFT: quadriceps rectus femoris thickness (mm). QRFA: quadriceps rectus femoris area (cm^2^). TTT: total thigh thickness (mm). PA: pennation angle.

**Fig. 2:**
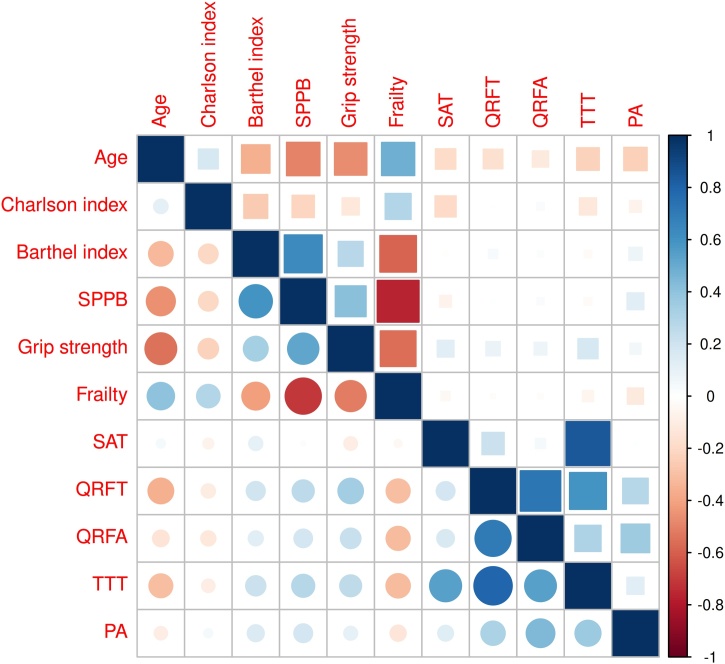
Heatmap showing the correlations between geometric muscle measurements and other study variables. Circles represent men and squares represent women. SPPB: Short Physical Performance Battery. SAT: Subcutaneous adipose tissue. QRFT: quadriceps rectus femoris thickness (mm). QRFA: quadriceps rectus femoris area (cm^2^). TTT: total thigh thickness (mm). PA: pennation angle.

Table 3 presents the results of the linear associations between ultrasound measurements and study variables, and Fig. 2 displays a heatmap of correlations between muscle geometric variables and other collected parameters. Age was negatively correlated with QRFT and TTT in both sexes, while SAT and PA showed negative correlations with age only in women. Associations with comorbidity were observed for SAT and TTT in women and TTT in men, likely reflecting the influence of adipose tissue. A slight association between dependency in BADL and QRFT was identified in men, but no such relationship was observed in women. Functional variables—including grip strength, frailty, and physical performance—were strongly correlated with QRFT, QRFA, and TTT in men. Additionally, PA demonstrated a significant association with SPPB scores in both sexes.

**Table 3:** Linear associations between muscle ultrasound measurements and different study variables.

	Age	Charlson	Barthel	SPPB	Grip strength	Frailty phenotype
SAT men	0.03 (−0.08 to 0.13)	−0.12 (−0.46 to 0.21)	0.04 (−0.03 to 0.11)	0.02 (−0.23 to 0.27)	−0.05 (−0.13 to 0.04)	−0.11 (−0.63 to 0.42)
SAT women	**−0.22† (**−**0.35 to** −**0.08)**	**−1.08† (**−**1.73 to** −**0.44)**	−0.01 (−0.09 to 0.07)	−0.15 (−0.44 to 0.14)	0.18* (0.00 to 0.35)	−0.18 (−0.75 to 0.39)
QRFT men	**−0.23‡ (**−**0.33 to** −**0.12)**	−0.21 (−0.55 to 0.13)	0.08* (0.01 to 0.15)	**0.39† (0.15 to 0.64)**	**0.17‡0.09 to 0.26)**	**−0.97‡ (**−**1.48 to** −**0.46)**
QRFT women	**−0.08† (**−**0.14 to** −**0.03)**	−0.00 (−0.27 to 0.26)	0.01 (−0.02 to 0.04)	0.01 (−0.11 to 0.12)	−0.05 (−0.02 to 0.12)	−0.02 (−0.25 to 0.21)
TTT men	**−0.49‡ (**−**0.74 to** −**0.23)**	−0.47* (−**1.31 to 0.38)**	0.23 (0.06 to 0.40)	**1.05† (0.44 to 1.65)**	**0.32† (0.11 to 0.52)**	**−2.47‡ (−3.73 to** −**1.21)**
TTT women	**−0.39‡ (**−**0.60 to** −**0.19)**	−1.06* (−**2.03 to** −**0.09)**	−0.03 (−0.14 to 0.09)	−0.10 (−0.52 to 0.32)	**0.37† (0.12 to 0.63)**	−0.38 (−1.22 to 0.46)
QRFA men	−0.04 (−0.09 to 0.01)	−0.11 (−0.27 to 0.05)	0.02 (−0.01 to 0.06)	0.13* (0.01 to 0.25)	0.05* (0.01 to 0.09)	**−0.46‡ (**−**0.70 to** −**0.22)**
QRFA women	−0.03 (−0.05 to 0.00)	0.03 (−0.10 to 0.15)	0.00 (−0.01 to 0.02)	0.01 (−0.04 to 0.06)	0.02 (−001 to 0.05)	−0.01 (−0.12 to 0.09)
PA men	−0.05 (−0.13 to 0.04)	0.06 (−0.19 to 0.32)	0.05 (−0.00 to 0.10)	0.21* (0.02 to 0.40)	0.04 (−0.03 to 0.10)	−0.33 (−0.73 to 0.07)
PA women	**−0.11‡ (**−**0.17 to** −**0.06)**	−0.14 (−0.40 to 0.12)	0.02 (−0.01 to 0.05)	0.12* (0.01 to 0.23)	0.03 (−0.04 to 0.10)	−0.22 (−0.44 to 0.01)

All data are B (95% CI). SAT: Subcutaneous adipose tissue. QRFT: quadriceps rectus femoris thickness (mm). QRFA: quadriceps rectus femoris area (cm^2^). TTT: total thigh thickness (mm). PA: pennation angle. * p < 0.05. † *p* < 0.01. ‡ *p* < 0.001.

## 4 Discussion

To our knowledge, this is the first study to present normative values for commonly used geometric ultrasound measurements of the quadriceps rectus femoris (QRF) at the mid-thigh in community-dwelling older adults. Establishing these normative values is essential for determining sex-specific cut-off points for conditions such as sarcopenia, frailty, malnutrition, and for predicting relevant outcomes like mortality, hospitalization, functional decline, or institutionalization. Additionally, we provide insights into the associations between these ultrasound measurements and other age-related variables, including comorbidity, physical function, dependency, strength, and frailty.

While normative muscle ultrasound values for community-dwelling older adults are scarce, comparisons with other populations can help validate our findings. For instance, a study of elite alpine skiers reported maximal anatomical cross-sectional QRF areas of 12.38 ± 2.67 cm² in females and 15.19 ± 3.13 cm² in males [22], approximately double the 80th percentile of our cohort. Similarly, a study on typically developing children aged 4–10.9 years found mean QRF areas of 5.38 cm² in boys and 5.32 cm² in girls, comparable to the 50th percentile of our cohort, while mean QRF thicknesses (QRFT), 13.14 and 12.81 mm respectively, were slightly lower than our mean values [23].

Our group published the results of the ECOSARC-1 study, conducted in 95 hospitalised older adults. Measurements were taken at distal 2/3 between anterosuperior iliac spine and superior patella border instead of mid-thigh, but mean values of both sexes for QRFT was 7.5 mm, and for QRFA 1.82 cm^2^, less than half of the 20th percentile of the ECOSARC-2 values. Patients in the ECOSARC-1 study presented higher age (mean 87.8 years), higher dependency in BADL (mean Barthel index 59), lower physical function (mean SPPB 3.1), and lower hand grip strength (mean 14.7 kg) [24].

Several studies have explored the use of muscle ultrasound in diagnosing sarcopenia [10,17,[25], [26], [27], [28]]. For example, de Luis Roman et al. examined the utility of QRF ultrasound measurements in hospitalized patients at risk of malnutrition. Although their findings showed fair diagnostic performance for sarcopenia in men only, their sample was not representative of community-dwelling older adults due to a younger mean age (58.5 years), lower BMI (22.9 kg/m²), and moderately low grip strength (25.0 kg). Their reported QRFA and QRFT values—4.09 ± 1.42 cm² and 11.10 ± 3.80 mm in men; 3.33 ± 1.13 cm² and 9.51 ± 2.89 mm in women—were below the 20th percentile of our cohort [26], highlighting the distinct characteristics of their study population and the potential utility of ultrasound in identifying malnutrition.

In another study conducted on Thai community-dwelling adults aged ≥60 years with sarcopenia, QRFT cut-off values of ≤11 mm for men and ≤10 mm for women yielded sensitivities and specificities of 90.9% and 92.2%, respectively [10]. These thresholds also fall below the 20th percentile of our participants' values. Furthermore, a study on ambulatory older adults (mean age: 76.8 years) demonstrated that QRFT had stronger associations with muscle strength and quality than bioimpedance-derived muscle measurements [25]. Finally, a meta-analysis concluded that muscle ultrasound demonstrates low-to-moderate diagnostic accuracy for identifying sarcopenia. However, it highlighted that combining muscle quality and quantity indicators could significantly enhance its diagnostic performance [11].

Last, a study in 91 community-dwelling older adults in Taiwan showed that US-derived RF muscle volume was accurate when predicting and diagnosing sarcopenia [27], and another study in 150 community-dwelling older adults with a mean age 80 years, QRFT showed good association with muscle mass and strength, highlighting the potential of US as a screening tool for sarcopenia [28].

Our study has several limitations and strengths. The primary limitation is that it is not population-based; however, we included participants from ten sites across Spain—seven hospital-based and three primary care settings—with a wide age range (70–99 years) and one-third female representation. All participants were ambulatory, and functional variables exhibited broad variability. Another limitation concerns the external validity of our findings; nonetheless, we believe that establishing normative values across diverse populations (e.g., Caucasian, Black, Hispanic, Asia-Pacific) is crucial for future comparisons. Meta-analyses combining such data may ultimately yield reliable cut-off points for clinical use. Methodological differences in acquisition points and ultrasound parameters pose challenges for direct comparisons with other studies; however, strict protocols like ours could facilitate harmonization in multicenter research efforts. The reliability of muscle ultrasound depends on operator expertise—a factor addressed in previous studies confirming its validity and reliability for assessing large muscle parameters in older adults [[29], [30], [31]]. In the ECOSARC-2 project, we implemented rigorous training protocols for sonographers with excellent results.

Among its strengths, this study is the first to provide normative values for community-dwelling older adults using a robust methodology applied across diverse clinical settings [21]. Sonographers underwent comprehensive training under expert supervision with concordance evaluations prior to data collection. All images were stored securely on the Clarius® cloud platform and reviewed for adequacy. The protocol received approval from ethics committees at all ten participating sites, and informed consent was obtained from all participants.

Our research group is committed to sharing data with other cohorts to advance the development of a multicenter database that could provide broader normative values across populations and establish cut-off points for sarcopenia and related conditions in older adults. Future challenges include analyzing muscle quality through video-based assessments of muscle contraction and conducting longitudinal follow-ups to evaluate adverse events. We believe this roadmap represents a critical step forward in advancing ultrasound-based sarcopenia assessment methodologies.

## CRediT authorship contribution statement

PA was the coordinator of the complete work, ELJ designed the work and contributed with data interpretation, drafting of the work, final approval of the version to be published, and agreement to be accountable for all aspects of the work in ensuring that questions related to the accuracy or integrity of any part of the work were appropriately investigated and resolved. PRP, AGR, MNA, AAS, MFGR, CAB, VCC, GMG, JRS, RAC, RRM, CPH, FZF, BDV, PLSG, IMM, RGM, RMC, NMV, EGJ, MSB, EBCZ, AAC, participated in data acquisition, drafting of the work, final approval of the version to be published, and agreement to be accountable for all aspects of the work in ensuring that questions related to the accuracy or integrity of any part of the work were appropriately investigated and resolved. FAP participated in the statistics, final approval of the version to be published, and agreement to be accountable for all aspects of the work in ensuring that questions related to the accuracy or integrity of any part of the work were appropriately investigated and resolved. All authors had a role in writing the final manuscript and approved the final version.

## Ethics statement

The experiments comply with the current Spanish laws.

## Declaration of Generative AI and AI-assisted technologies in the writing process

During the preparation of this work the authors used Perplexity in order to improve language editing. After using this tool/service, the authors reviewed and edited the content as needed and take full responsibility for the content of the publication.

## Funding

This work was supported by CIBERFES, Instituto de Salud Carlos III, Ministerio de Economía y Competitividad, España, Ayuda cofinanciada por el Fondo Europeo de Desarrollo Regional FEDER Una Manera de hacer Europa (Grant number CB16/10/00408).
